# Emotions on Twitter as crisis imprint in high-trust societies: Do ambient affiliations affect emotional expression during the pandemic?

**DOI:** 10.1371/journal.pone.0296801

**Published:** 2024-03-05

**Authors:** Marina Charquero-Ballester, Jessica Gabriele Walter, Astrid Sletten Rybner, Ida Anthonj Nissen, Kenneth Christian Enevoldsen, Anja Bechmann

**Affiliations:** 1 Department of Media and Journalism Studies, School of Communication and Culture, Aarhus University, Aarhus, Denmark; 2 Interacting Minds Centre, Aarhus University, Aarhus, Denmark; La Trobe University - Melbourne Campus: La Trobe University, AUSTRALIA

## Abstract

During the Covid-19 crisis, citizens turned to Twitter for information seeking, emotional outlet and sense-making of the crisis, creating ad hoc social communities using crisis-specific hashtags. The theory of ambient affiliation posits that the use of hashtags upscales the call to affiliate with the values expressed in the tweet. Given the deep functional tie between values and emotions, hashtag use might further amplify certain emotions. While emotions in crises-hashtagged communities have been previously investigated, the hypothesis of amplification of emotions through hashtag use has not yet been tested. We investigate such effect during the Covid-19 crisis in a scenario of high-trust Nordic societies, focusing on non-hashtagged, crisis hashtagged (e.g., ‘#Covid-19’) and threat hashtagged (e.g., ‘#misinformation’) tweets. To do so we apply XLM-RoBERTa to estimate Anger, Fear, Sadness, Disgust, Joy and Optimism. Our results revealed that crisis-hashtagged (#Covid-19) tweets expressed more negative emotions (Anger, Fear, Disgust and Sadness) and less positive emotions (Optimism and Joy) than non-hashtagged Covid-19 tweets for all countries except Finland. Threat tweets (#misinformation) expressed even more negative emotions (Anger, Fear, Disgust) and less positive emotions (Optimism and Joy) than #Covid-19 tweets, with a particularly large effect for Anger. Our findings provide useful context for previous research on collective emotions during crises, as most Twitter content is not hashtagged, and given the faster spread of emotionally charged content, further support the special focus on specific *ad hoc* communities for crisis and threat management and monitoring.

## Introduction

During the Covid-19 pandemic, social media became important arenas for information seeking and communication as well as individual and collective coping, due to prolonged lock-down periods that impeded physical interaction. As a result, social media‘s role as important sources for understanding crises and their imprints through collective emotional expressions became even more pronounced. We know little of whether and how emotional expressions vary depending on their conversational context and the differentially threatening nature of such contexts [1] during a crisis. Studies on collective emotions in nations around the world predominantly use crises-hashtags to understand crises imprints, thus, using a specific conversational context [2–4]. However, non-hashtagged content and hashtags that refer to further threats (here illustrated by misinformation hashtagged tweets) are also provided during crises and may provide different grounds for using social media for studying crises and related threats imprints.

In February 2020, only a month after the Covid-19 pandemic was declared, the World Health Organization (WHO) announced that the health crisis was accompanied by yet another threat: a strong prevalence of misinformation as characteristic of the ‘infodemic’ (WHO 2020). The WHO defined infodemic in one of the Covid-19 reports as "an overabundance of information—some accurate and some not—that makes it hard for people to find trustworthy sources and reliable guidance when they need it” [5]. Beyond the direct impact on health, due to the adoption of harmful behaviours, the WHO contemplates mistrust and undermining of social cohesion as some of the effects of the infodemic. Thus, they highlight the need to understand misinformation as an additional threat. Its social media logics would then be part of crises imprints for developing and maintaining trust in health authorities, health service delivery and public health response, as well as in science and the media. Furthermore, misinformation is potentially harmful by affecting health-protective behaviour [6], therefore posing an additional threat within the health crisis. Although previous public health crises have also affected millions, constant media coverage regarding COVID-19 and extended use of social media endowed this global health crisis with an additional character of emergency that can also be expected in future crises. Misinformation reaches more people and spreads further than true information while also negative emotional expression fuels spread [7–10].

To date there is a large body of research exploring emotional expressions on Twitter, mostly in the form of sentiment analysis looking at positive, negative or neutral sentiment, but a comprehensive overview is still lacking. Previous research has shown that negativity is amplified by Twitter algorithms [11] which could explain to some degree why emotions such as Anger and Disgust [2] appear to be predominant. The reason that some emotions are more predominant than others can be due to the platform ecosystem, and so our focus is not on the absolute expression of specific emotions but rather on the relative amplification depending on the conversational context.

Going beyond social media into survey studies, research has highlighted Fear and Anxiety as some of the main emotional responses in the population during the Covid-19 crisis [12–15], while Twitter studies have also observed signs of this Fear and Anxiety [16–18]. One of these studies found furthermore that public emotions shifted strongly from Fear to Anger, with Sadness and Joy also coming to the surface, over the course of two months at the beginning of the pandemic [16]. However, these studies were conducted on data from mixed geolocations [16, 19] or from countries where less resilience would be expected in the face of a crisis due to, for example, their lower indices on trust in the government and the media [18]. It is also worth noting that most of these studies address earlier stages of the Covid-19 crisis, while little is known about the emotional responses at later stages of the pandemic, when the initial coping mechanisms have been exhausted due to the population’s resilience having been probed for months. Previous research shows strong correlations between survey data on positive and negative feelings and estimations through machine-learning approaches [17] indicating added values of Twitter studies such as the present one, with the further advantage of allowing for distinction between three conversational contexts.

We focus on the four largest Nordic countries (Denmark, Finland, Norway and Sweden, here referred to as the Nordic countries). Nordic countries share characteristics such as being healthy democracies [20], comprehensive healthcare systems [21], high levels of education [22] and high levels of trust in organizations and the media [20, 23]. These characteristics render them an ideal case for stabilising potential societal trust issues that can affect the collective emotional imprint on Twitter.

The Nordic countries being resilient democracies with high-trust societies is particularly important for our study. This is because if we find strong emotional imprints in those countries, we could expect even stronger imprints in less favourable contexts. In democratic societies, public support is an essential pre-requisite for the introduction of new policies, explaining why politicians are often reluctant to introduce policies with low support from the public [24, 25] as it would otherwise damage governmental support. Previous research supports a positive effect of trust on acceptance of public policies [26–28]. However, more recent research has highlighted the lack of direct effect of trust on policy acceptance, arguing that any effect of trust is actually mediated via emotions [29]. In other words, general trust in the government, which in the case of the Nordic countries is a unifying factor, can influence affective and cognitive responses to specific policies and management strategies. A further study investigated how public trust in governmental institutions for addressing the COVID-19 crisis affects individuals’ risk information-seeking and avoidance and found that trust was negatively related to Fear, Anger, Sadness and Anxiety, and positively related to Hope [30]. In a context where we are seeing a general decline in trust across EU member states [31], the study of those emotions in the highest trusting societies becomes paramount for improving crisis monitoring and management.

Here, we examine the emotional expressions of citizens in high trust societies, if and how they differ in the conversational contexts on Twitter (non-hashtagged, Covid-19 hashtagged, and misinformation hashtagged tweets) in order to evaluate such emotional expressions as crises imprints for future crises and threat management and monitoring. Of note, we consider both the non-hashtagged and the hashtagged tweets related to the Covid-19 crisis, given that the pandemic permeated almost all aspects of life and that studies show that most users do not use hashtag in tweets [1].

### Theoretical framework: Emotional expressions online as crises imprints

#### Analyzing emotions on Twitter computationally

Most studies analyzing emotions as a crisis imprint on Twitter use a sentiment analysis approach, based on a dimensional conceptualization of emotion, where emotions are assigned a score according to their positive, neutral or negative valence [32, 33]. Yet, such an approach does not use a more complex understanding of emotions by distinguishing between different kinds of negative and positive emotional expressions that could have an impact on the understanding of crises. In order to distinguish between different emotions, we need a categorical approach more in line with the theories of emotion proposed by Izard [34] and Ekman [35] that are still currently applied to social media data [36–39]. These theories argue in favour of the existence of a set of basic, universal human emotions produced by an innate hardwired neuromotor program, that allows for building and explaining all other emotions.

Adopting the categorical approach allows us to have easily understandable affective labels (e.g., Anger, Fear, Sadness) that might be more interpretable and useful for the purpose of crisis monitoring than sentiment analysis. While categorical emotion detection in short texts is methodologically more challenging, recent development of powerful and multilingual machine learning models e.g., [40] has facilitated detection of such specific emotions from text data in lower resourced languages, opening up new possibilities regarding the study of emotions on social media.

#### Emotional expression in language: Between human feelings and collective crisis imprint

While there is a robust body of research on emotions from a psychological perspective, with studies based on both–a dimensional or categorical–approach, there is currently no unified theory of affect in language, or even more specifically in our case, short written language on social media. Instead, we find a broad range of approaches to the expression of emotion in general. The reason for this may be the complex relationship between emotion and language; i.e., those feelings might be at different levels of consciousness and intentionality, being more or less explicit in language, and may relate to various aspects of the communicative context [41]. While this limits the accuracy with which we can estimate the emotion of a specific individual through the text they publish on social media, the aggregation of emotional expressions extracted from tweets in a certain context and at a certain time holds value for the study of collective emotions. In support of this claim, we can find numerous examples of studies leveraging Twitter data to extrapolate valid conclusions about collective emotions [3, 17, 42, 43]. We refer to collective emotions as emotions that are experienced and expressed by a group or community of individuals. These emotions arise as a result of a shared experience, values, beliefs, or events, in our case a specific time period of the Covid-19 crisis and the additional threat concerning misinformation.

#### Ambient affiliation: Emotions in different conversational contexts on Twitter

Building on the research that shows that social media emotion macroscopes are a valid reflection of society’s emotional experiences, we adopt a framework based on appraisal theory [44] and developed specifically around Twitter communication by Zappavigna [45–47]. Zappavigna posits the term ‘ambient affiliation’ arguing that in addition to their topic-marking function, Twitter hashtags act as linguistic markers indicating the target of the appraisal in the tweet, which can be used to upscale the call to affiliate with the values expressed in the tweet. Importantly, values and emotions are fundamentally connected, being both psychological markers of subjective relevance. According to the psychological appraisal theories of emotion [48], emotions arise when value concerns are at stake; according to theories of value, a value that is threatened or supported gets imbued with feelings [49]. Thus, values appear to be antecedents of emotions when emotional experiences arise in response to value-relevant stimuli, as shown by Conte and colleagues [50].

In the following we illustrate this idea with an example from Zappavigna’s work. We take the following tweet accompanied by a hashtag: ‘From fear, hatred and economic collapse to hope, search for common ground and prosperity again. Good change! #obama’. Here, the hashtag, renders the tweet more ‘searchable’ and in so doing, intensifies the ‘call’ to affiliate with the values in the tweet. Zapavigna and colleagues argue that the presence of the hashtag, when compared with the non-hashtag example, expands the potential meaning of the tweet by making the coupling more available for search and more likely to be automatically followed by those subscribing to this tag, hence, becoming ‘louder’, ‘hyper-charged’, and making it more ‘bondable’. The tweet also conveys the emotions associated with the underlying values of the user, arguably making them an essential part of that ‘hyper-charged’ communication. Being searchable opens up a new kind of sociality where microbloggers engage in ambient affiliation. The affiliation derives from the social function of the hashtag, which by grouping tweets creates *ad hoc* social communities or publics [1]. This affiliation is ambient in the sense that the users may not have interacted directly and likely do not know each other, and may not interact again. As such, Twitter offers a medium for expressing personal evaluations to a large body of listeners with which one can affiliate ambiently. This way of interacting is a relatively new cultural process. We tie in this concept of ambient affiliation with collective emotions in a similar fashion to Rimé [51] who suggested that chain reactions in response to sharing about a collective event would contribute to the construction of an emotional climate. A table with definitions of the key concepts presented can be found in **S1 Table**.

Our focus on the emotional expressions of citizens in Nordic high trust societies, if and how they differ in the conversational contexts on Twitter (non-hashtagged, Covid-19 hashtagged, and misinformation hashtagged tweets) helps to understand the impact of hashtag use on emotional expression and the possibility for people to channel specific emotions into additional threat contexts like misinformation.

## Materials and methods

First, we aim to isolate the effect of ambient affiliation on emotional expression by comparing emotional scores of non-hashtagged tweets from a timeframe within the pandemic with tweets containing one or more hashtags related to Covid-19. We hypothesize that placing the emphasis on Covid-19 through the use of that hashtag would be related to increased intensity in emotional expression. Second, we compare the intensity of emotional expression between two spaces in which ambient affiliation might be occurring, as inferred through the use of two clusters of hashtags, one specifically addressing the Covid-19 crisis and another one around the problem of misinformation and fake news. This second comparison will allow us to investigate whether, in our given context of high-trust, an additional threat (i.e., misinformation) is used to outlet specific emotions like Anger using hashtags to create ambient affiliation around them. We set no restrictions on the primary emotions evaluated, which allowed us to evaluate the effects on Anger as well as a broader range of emotions such as Fear and Sadness, and the interactions between them.

We started by collecting a very large dataset using most-differentiated stop words in the four different Nordic languages that are included in this study, reaching a total of 57.8 million tweets spanning the time period between August 2020 and March 2021. We chose this time period, pertaining to the second wave of the pandemic, because we were interested in the emotional expressions that would appear once the crisis has had time to erode the resilience of the population. In regards to the relationship between high-trust and the implementation of new policies, looking at the second wave of the pandemic also ensured that governments had had time to develop and adopt those policies aimed at dealing with the crisis. Using the resulting country-specific datasets, we conducted correlational analyses to rule out potential correlations between emotional profiles and demographics of the number of deaths specific to any of the countries (see **S1 Fig**). We then extracted the subsamples (for details, see Hashtag extraction in **S1 Appendix** on 1) tweets lacking any hashtags (referred to as ‘non-hashtagged’) 2) tweets containing the most often used Covid-19 hashtags (referred to as ‘#Covid-19’, see **S2 Table**) and 3) tweets containing hashtags related to an additional threat, namely misinformation (referred to as ‘#misinformation’, see **S3 Table**). Using the created subsamples we addressed our specific hypotheses, analysing first the effect of hashtags on emotional expression and then the channelling of emotions towards the additional threat in the high-trust context of the Nordic countries.

**Fig 1:**
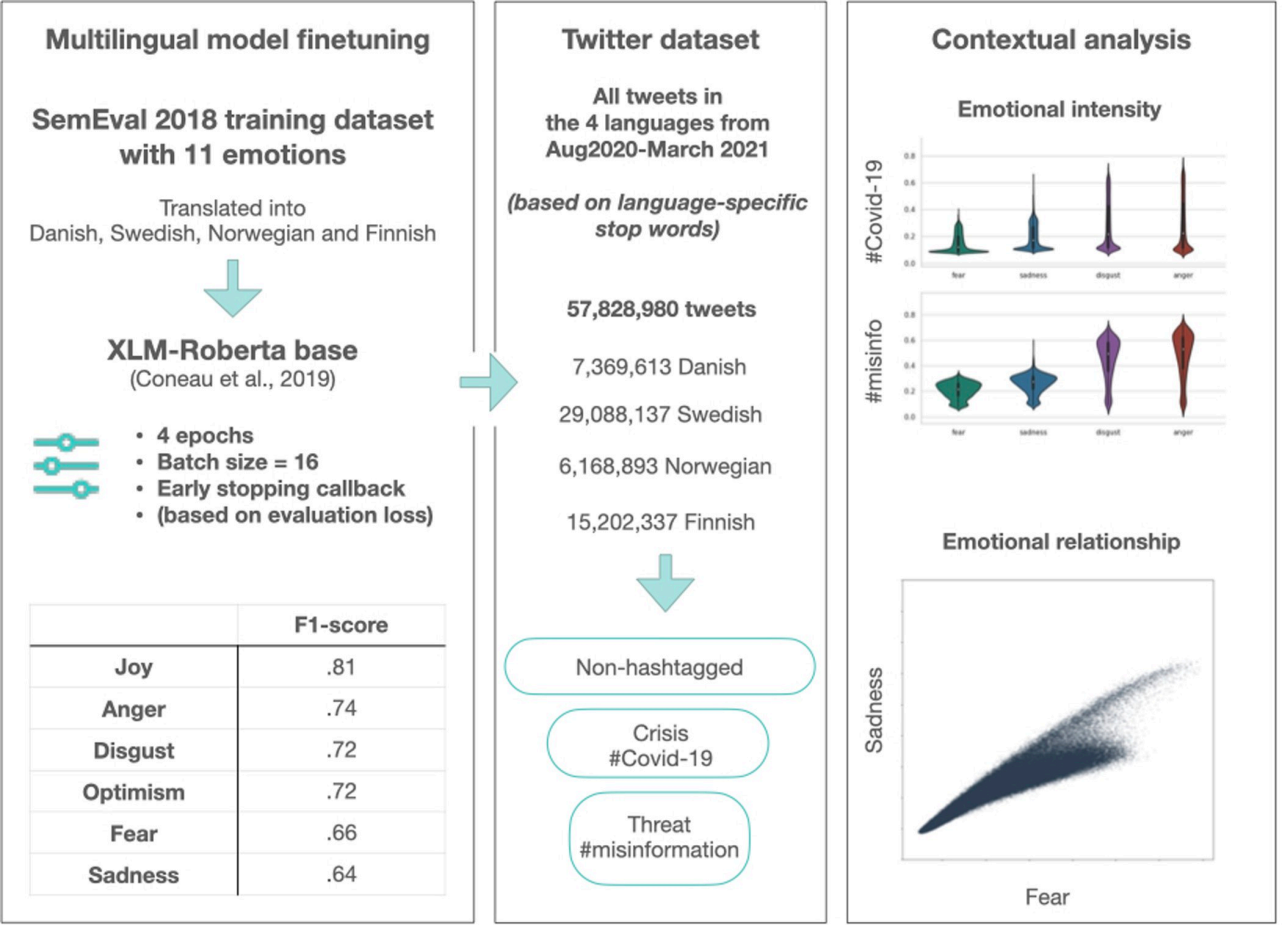
Overview of the analysis pipeline. Left: XLM-RoBERTa base was fine-tuned using the SemEval 2018 training dataset (subtask 5) translated into the Nordic languages present in our data. We filtered for emotions that obtained an f1-score of above .6 as a minimum standard for model performance. Middle: Data was scraped for the time period between August 2020 and March 2021 based on language specific stopwords, reaching a total of 57,828980 tweets. From that sample we extracted 1) a sample of tweets not containing hashtags (non-hashtagged) 2) a #Covid-19 subsample 3) a #Misinformation subsample. Right: Overview of emotions in the data in order to characterise the Nordic Twittersphere and statistical analyses to compare non-hashtagged vs. #Covid-19 and #Covid-19 vs. #Misinformation.

First, we analysed a large Twitter dataset containing tweets in four of the Nordic languages: Danish, Norwegian, Swedish and Finnish using a set of stop words in each of the languages to scrape through the Twitter API. The sampling method aimed at collecting as many tweets as possible without any hashtag or Covid-related keyword specifications, allowing for an overall sample representative of the Nordic Twitter during Covid-19, as well as the possibility to filter it down for the creation of subsamples **(Fig 1).** For details on the use of stop words for data collection, see **S1 Appendix**. Our dataset includes tweets that were posted between August 2020 and March 2021. The Twitter dataset contained a total of 57,828,980 tweets (29,088,137 in Swedish, 6,168,893 in Norwegian, 7,369,613 in Danish, and 15,202,337 in Finnish) **(Fig 1)**. Data collection and data analysis complied with Twitters’ terms and conditions. Data preprocessing included removing mentions, URLs and decoding emojis present in the text using Demoji 1.1.0.

We used XLM-RoBERTa base model proposed by Conneau and colleagues https://huggingface.co/xlm-roberta-base [40], based on the initial RoBERTa model released in 2019 [52]. A comparative study looking at sentiment classification effectiveness across 4 different models, established that BERT-based models were superior to all other models in all effectiveness measures (Accuracy, precision, recall, F1-score) [53]. The model shares the advantages of traditional transformer models, which use attention mechanisms to extract relational context and even long-range dependencies in a sentence. The attentional mechanisms of this models have been shown to assign specific attention heads to linguistic notions of syntax, such as direct objects of verbs, determiners of nouns and objects of prepositions [54]. Contextual embeddigns of the model incorporate intensifiers and negations, meaning that the model can learn those from the training data. A factor that can affect the performance of the model in handling intensifiers and modifiers, is the size of its training data. However, this particular model, was pretrained on 2.5TB of filtered CommonCrawl data. It was furthermore fine-tuned on domain-specific data, which can further help improve its performance in recognizing and handling these linguistic elements effectively. Finetuning parameters for the XLM-RoBERTa model were as follows: learning rate = 2e; batch size = 16, number of epochs = 4 (with early stopping call-back based on the evaluation loss) **(Fig 1).**

To fine-tune the model for emotion detection, we used the SemEval 2018, task 1 [55], containing labels for 11 emotions (for more details, see Training data in **S1 Appendix**). Being a multilingual model, it allowed us to use the same model to analyse all tweets independently of their language. Since we needed the training dataset to be available in the four Nordic languages of this study, we translated the English version into all four Nordic languages using the eTranslation tool provided by the European Commission (https://ec.europa.eu/info/resources-partners/machine-translation-public-administrations-etranslation_en). To assess the accuracy of the AI-based translation, we asked native speakers to rate the accuracy according to two criteria: Meaning and emotionality. Each of the criteria could be assigned a score ranging from 1–3, representing 1) poor accuracy, 2) fair and 3) good accuracy. Averaging across languages, we obtain a score of 2,424 for Meaning and a score of 2,615 for Emotionality out of a maximum of 3 (for more details, see **S4 Table**), placing the accuracy of the translation between fair and good according to our scale.

For evaluation of the fine tuning on the validation set, a threshold of .5 was set above which a tweet was considered to have been assigned to a specific emotion category according to the model. We then evaluated the performance of the model on the validation set focusing primarily on the f1-score, choosing this metric due to our imbalanced dataset. Performance metrics after the finetuning can be found in the Supporting Information (**S5 Table**). As shown in the Supplementary material, only five out of eleven emotions obtained an f1-score above .6, which we set as the threshold above which we consider the model to be able to reliably detect the emotion. These were Anger, Disgust, Fear, Joy, Optimism and Sadness.

We then estimated the probabilities for each emotion to be present in each tweet. Tweets with all emotional probabilities below .5 were considered neutral tweets and excluded from further analysis. We separated original tweets from retweets and extracted non-hashtagged, Covid-19 hashtagged and misinformation hashtagged tweets as detailed in the Supporting Information (**S1 Appendix** and in **S2 and S3 Tables)**. Statistical analysis comparing two samples of emotion probabilities were carried out through two-sided permutation as implemented in R. Significance level was set at α = 0.05. Additionally, Cohen’s d standardized effect sizes [56] are reported to measure the extent of the difference between each pair of group means. Further documentation and code related to the study can be found in our teams’ github.

Due to the sensitive nature of the data, we were unable to make a public release. However, GDPR compliant researchers can access the tweet ID’s files for the three subsamples here.

## Results and discussion

### The effect of ambient affiliation through hashtag use on emotional expression

Our findings in regard to the use of hashtags for ambient affiliation and its effect in emotional expression reveal that, in the majority of countries under study, #Covid-19 tweets contain more negative emotions (Fear, Disgust, Anger, Sadness) and less positive emotions (Joy and Optimism) than non-hashtagged tweets.

Averaging across countries and including all emotions, we observe similar distributions between the ‘non-hashtagged’ and the ‘#Covid-19’ subsample **(S2 Fig)**. The figure shows that most tweets were assigned a low score on the negative emotions (Fear, Sadness, Disgust, and Anger). In regard to the positive emotions Joy and Optimism, the plots show that the distribution of tweets containing low or high scores on these emotions are more comparable, having similar amounts of tweets in the lower and higher end of the distributions. The #misinformation tweets, on the other hand, appear to be quite different to the non-hashtagged and #Covid-19 tweets. Here we can observe that most tweets were assigned relatively high scores in Disgust and Anger, moderate scores in Fear and Sadness, and low scores on Joy and Optimism. Interestingly, we found that Fear did not appear as a driving emotion in the non-hashtagged and #Covid-19 tweets. Even though the non-hashtagged tweets appear to have more tweets with a low to moderate score for Fear compared to the #Covid-19 tweets, Fear appears to dominate more in the #misinformation tweets than in any of the other two conditions.

**Fig 2:**
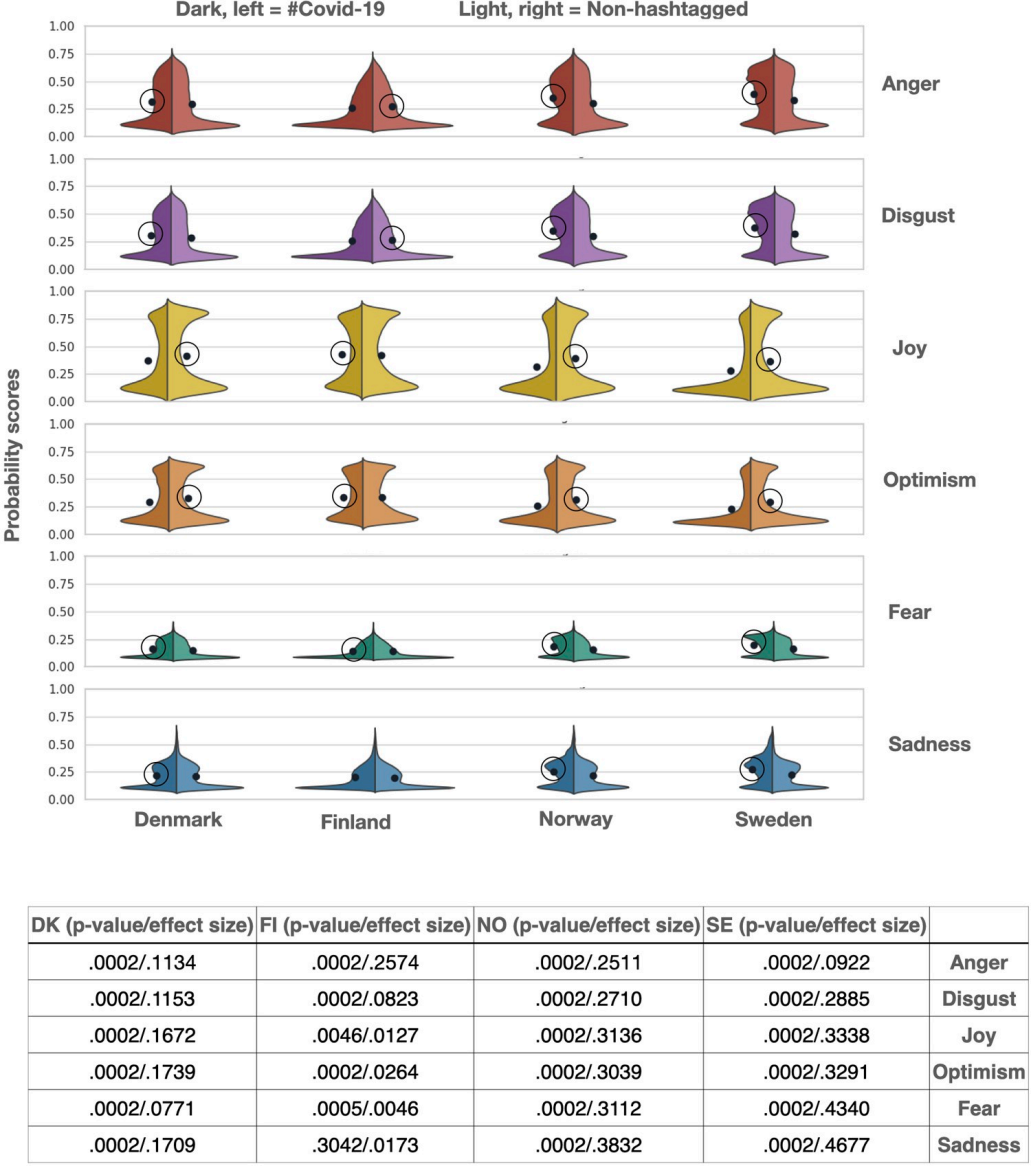
Comparison of emotional expression in #Covid-19 vs. non-hashtagged tweets. Negative emotions are significantly higher in #Covid-19 tweets than in the non-hashtagged tweets, with the exception of Finland (p < .005). Positive emotions are significantly lower in the #Covid-19 tweets than in the non-hashtagged tweets, with the exception of Finland (p < .005). However, the effect sizes are moderate to small throughout comparisons.

In order to get a deeper understanding of differences in emotional expression in #Covid-19 tweets in contrast to non-hashtagged tweets, we conduct analyses for all the emotions on the country level. Before diving into the specifics of these results, it is worth noting that we found no correlation between emotion scores and number of deaths per country, ruling out pandemic demographics as a driving factor for emotional expression in any of the countries **(S1 Fig)**. Focusing on the negative emotions in #Covid-19 in contrast to the non-hashtagged tweets, the most consistent finding across countries concerns Fear, which had higher probability scores in #Covid-19 tweets for all countries (Danish p = .0002; Swedish p = .0002; Norwegian p = .0002, Finnish p = .0002) (**Fig 2**). The probability scores for Sadness were also consistently higher in #Covid-19 tweets (Swedish p = .0002; Norwegian p = .0002, Danish p = .0002), except for the Finnish data (p = .3042), which showed no significant differences between #Covid-19 and non-hashtagged tweets. Disgust and Anger showed again consistent results across countries with the exception of the Finnish data; in this case, Disgust and Anger were significantly higher in the Danish (p = .0002), Norwegian (p = .0002) and Swedish (p = .0002) sample for #Covid-19 tweets, but significantly lower for the Finnish subsample (p = .0002).

In positive emotions we found opposite patterns. For example, Joy in the non-hashtagged tweets showed higher probability scores (Swedish p = .0002; Norwegian p = .0002, Danish p = .0002) except for Finland in which Joy was significantly higher in the #Covid-19 tweets (p = .0046) (**Fig 2**). Optimism was similar, with the non-hashtagged tweets showing higher probability scores (Swedish p = .0002; Norwegian p = .0002, Danish p = .0002) except for Finland showing significantly lower probability scores for this emotion (p = .0002). It is worth noting, however, that the effect sizes for the differences range from small to moderate, for both positive and negative emotions.

Overall, these results suggest that in the context of a crisis in high-trust societies, the use of crisis-related hashtags is often accompanied by an amplification of negative emotions and a reduction in positive emotions and thus seems to suggest that negative emotional expressions during crises might be used to propitiate ambient affiliation with *ad hoc* communities. Examples of tweets, edited to protect the privacy of the study participants, with high overall scores in emotionality, are provided for both subsamples in **S6 Table**.

Our results suggest that Twitter users seeking to bond through ambient affiliation in the context of a crisis appeal to negative emotions instead of aiming to amplify the positive ones in the situation with emotions such as Optimism and Joy. In fact, the expression of these two emotions was lower in the #Covid-19 than in non-hashtagged tweets for all countries except Finland. While our results seem to indicate that Finland is an exception in which citizens may amplify positive emotions in the context of a crisis in order to increase bonding, it is difficult to pinpoint whether this result might be best explained through linguistic, cultural or digital culture differences with the other Nordic populations. A potential explanation is that Finns might have chosen to remain more ‘silent’ (expressing less their Anger) in a specific Covid-19 context, as it has been shown that in Finland, silence is tolerated and in certain social scenes it is preferred to small talk [57, 58], which might extend to what might have been considered excessive and repetitive negative talk online regarding the crisis.

Interestingly, and contrary to the findings from other studies in which Fear and Anxiety appeared to drive a considerable part of emotional expression in relation to Covid-19, the scores estimated by our model were low **(Fig 2)**. This finding could be related to multiple factors; most importantly, the Nordic countries in the comprehensiveness of their welfare system, the high-trust of its populations and the relatively lower impact of the Covid-19 crisis, may at least partly explain this finding. Another potential explanation is that the time period chosen in this study was not at the beginning of the crisis, when many of the studies with data from other regions were conducted, but during the second wave of the pandemic. Lower levels of Fear might be the result of more information being available and the restrictive measures being already normalised among the populations. Diving deeper into the potential amplification or dampening of Fear expression in relation to ambient affiliation, we found however significantly more Fear expression in the #Covid-19 than in the non-hashtagged tweets. This suggests that, in the context of the Covid-19 crisis, Twitter users ‘appeal’ to Fear in order to bond with other users and become louder in the community even if fear is not the predominant emotion. However, as mentioned above, this effect was not specific to Fear but rather present in all negative emotion.

### The effect of different ambient affiliation contexts on negative emotions

**Fig 3:**
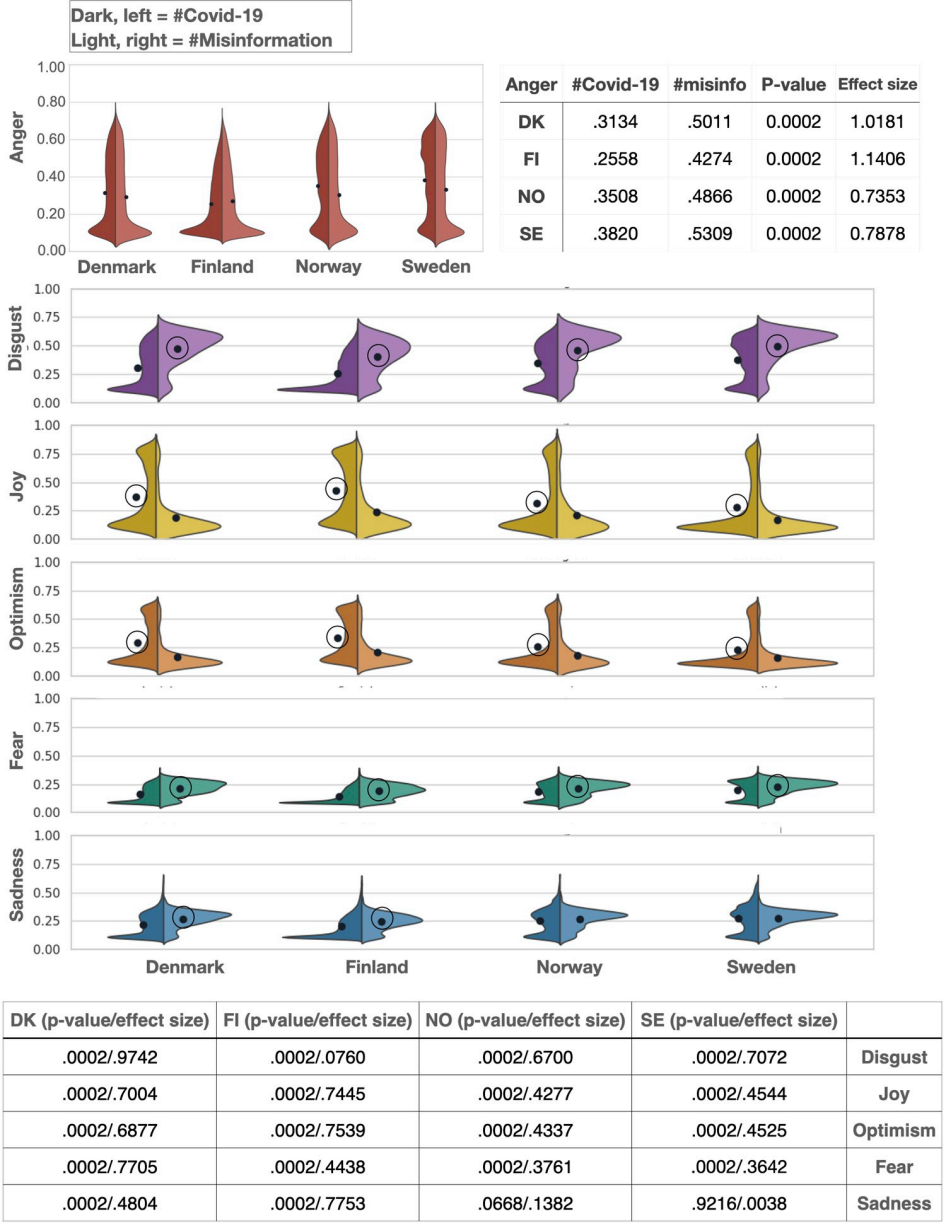
#misinformation holds more anger than #Covid-19. We found significantly more Anger expression in the #misinformation than in the #Covid-19 tweets, with moderate to high effect sizes across countries. Negative emotions like Disgust and Fear were also consistently higher in the #misinformation vs. #Covid-19 tweets, although the effect sizes here were smaller than for Anger and ranging from small to large across countries and emotions. Positive emotions were higher in the #Covid-19 than in the #misinformation subsamples, with small to moderate effect sizes.

Next, we address our second hypothesis concerning whether high-trust societies might channel some emotions towards additionally threatening conversational contexts (#misinformation). Analyses comparing the #Covid-19 to the #misinformation subsamples reveal significantly more Anger expression in the #misinformation than in the #Covid-19 tweets. Here the effect sizes are large in all four countries, ranging from 1.018 in Denmark to .735 in Norway (**Fig 3**). While other negative emotions like Disgust and Fear were also higher in the #misinformation than in the #Covid-19 tweets (**Fig 3**), the effect size was consistently highest for Anger across countries. Examples of tweets, edited to protect the privacy of the study participants, with high overall scores in emotionality are provided for both subsamples in **S7 Table**.

These analyses also revealed that emotions such as Joy and Optimism were consistently lower in #misinformation than in #Covid-19 tweets across the Nordic countries (p = .0002). Furthermore, we found that Sadness did not show a consistent effect across countries, since it was higher in #misinformation than in #Covid-19 tweets but only for Denmark and Finland (p = .0002). No significant differences between the two conditions were found in the Norwegian and Swedish data (p = .06 and p = .92) (**Fig 3**). Hence, when zooming into our second hypothesis regarding further amplification of Anger in a threat context (#misinformation) in contrast to the crisis context (#Covid19), we find a strong increase in Anger accompanied by a decrease in positive emotions, supporting our expectations.

While other negative emotions were also higher in the #misinformation than in the #Covid-19 subsamples, the difference in Anger had the largest effect size across countries. The largest increase in Anger in contrast to the other emotions might suggest that indeed there might be an amplification of Anger expression in additional threat contexts, perhaps even related to the channelling of difficult emotions. Disgust and Fear, two emotions closely related to Anger, were also consistently higher in the #misinformation than in the #Covid-19 subsamples across countries. Hence, the crisis and threat conversational contexts might be used as spaces for channelling negative emotions which might have different implications at the collective and at the individual level. While at the individual level the outlet of negative emotions might help with coping in a difficult situation [59, 60], at the collective level, and in addition to the amplifying effect of negative emotions by social media algorithms [7, 11], it can be harmful for crisis mitigation. This calls for future studies on the actual gain that societies can extract from participating in such *ad hoc* communities in regards to channelling of individual emotions.

**Fig 4:**
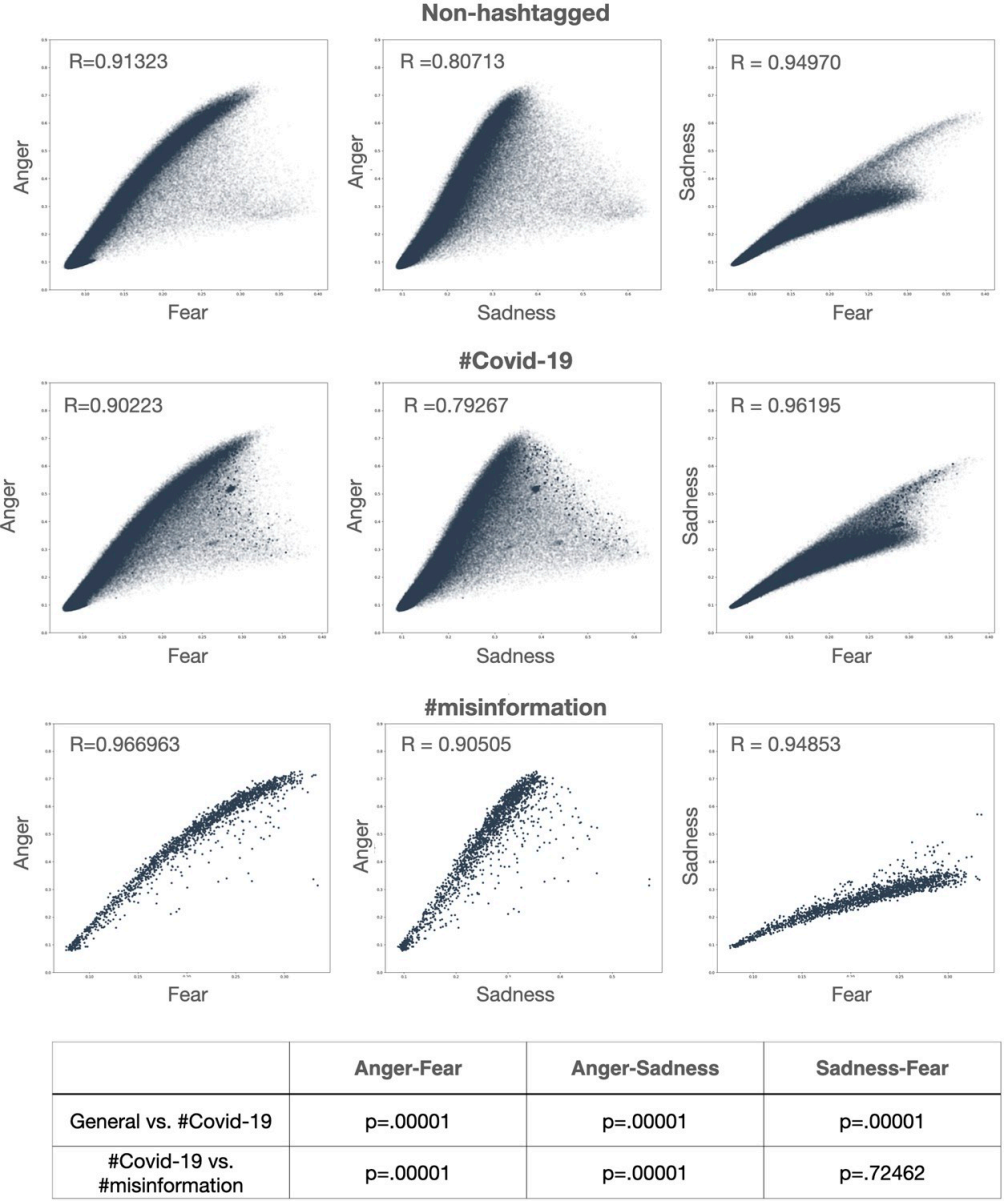
Negative emotion interactions in the three subsamples. a, b) In #Covid19 tweets, the correlation between Anger and Fear, as well as between Anger and Sadness, is weaker than in non-hashtagged tweets (p = .00001 for both). On the contrary, the correlation between Sadness and Fear is stronger in the #Covid than in the non-hashtagged tweets. b,c) The correlation between Anger and Fear, as well as between Anger and Sadness is stronger in the #misinformation than in the #Covid-19 subsample (p = .00001). No significant differences were found in the correlations between Sadness and Fear (p = .72462).

Last, we address the relationship between Anger and other negative emotions in the three conditions. We find strong linear relationships between all combinations of negative emotions and across all three conditions when combining the data for all countries **(Fig 4)**. When considering the slope of the three combinations, we noticed that the slope is steeper for the combination Anger-Fear and Anger-Sadness than for Sadness-Fear. In other words, it seems like high expression of Anger precludes high expression of Sadness or Fear, as if Anger was ‘placing a cap’ on the expression of these two other emotions.

When comparing those linear relationships across the three different conditions, we find significant differences in the strength of that relationship specific to the *ad hoc* community **(Fig 4)**. When looking at the relationship between Anger and Fear, we find the strongest correlation in the #misinformation subsample, being significantly higher in comparison to the non-hashtagged and the #Covid-19 tweets (p = .00001). The relationship between Anger and Sadness is also strongest in the #misinformation tweets, again significantly stronger in contrast to the non-hashtagged and #Covid-19 tweets (p = .00001). However, the relationship between Sadness and Fear is strongest in #Covid-19. In specific, it was significantly stronger in the non-hashtagged vs. the #Covid-19 (p = .00001). No significant differences were found in the #Covid-19 vs the #misinformation tweets (p = .72462).

Given that anger is the shared factor among the higher slope combinations, we suggest that this may be drawn back to a fundamental feature of the emotion of Anger in a way in which high expression of Anger limits the expression of Sadness or Fear. The underlying reason for this may be related to the behavioural consequences of Fear and Anger. While both fear and anger are high-arousal emotions, in contrast to sadness being a low arousal emotion [61, 62], the behavioural consequence of Anger is often action while the behavioural consequence of fear is avoidance [63, 64]. As these two behaviours are incompatible, the two emotions cannot coexist at high levels of intensity.

## Conclusion

In this study, we investigate emotions expressed in the Nordic Twittersphere during the second wave of the pandemic, due to our interest in the emotional responses from high-trust societies across three conversational contexts Twitter (non-hashtagged, crisis hashtagged, and additional threat hashtagged tweets). The three conversational contexts allow for a more comprehensive and contextualised understanding of emotions as crises imprints, essential for accurate crisis monitoring and effective management. In this context, we are particularly interested in negative emotions and even more specifically, in Anger, given the accentuating factor of additional threats in a crisis context and its potential to make societies spiral out of control. The study was framed around the concept of ‘ambient affiliation’ coined by Zappavigna [46] and explained in detail in the Introduction. Briefly, the concept refers to Twitter hashtags being used to upscale the call to affiliate with the values expressed in the tweet. Hence, here we look into the expression of emotions in high-trust societies in relation to crisis and additional threats, but with a special focus on the interplay between emotional expression and ambient affiliation. To our knowledge there are no large-scale studies investigating this interaction between emotional expression and the amplifying effect of hashtag use around societal distress in different conversational contexts.

The findings of our research have many relevant implications for various societal aspects, ranging from social dynamics to policy and decision-making. Here we take the expression of negative emotions as a sign of distress of the population under crisis. Understanding the collective negative emotions related to that distress can bring valuable insights into the dynamics of online communities, specially within the framework of ambient affiliation. In particular, our research brings new information into how negative emotions, in particular Anger, intensify in the presence of additional threats in high-trust countries. Furthermore, negative emotions, such as Anger and Fear, can fuel social unrest, conflicts, and divisions within communities. While we aimed at a comprehensive account of emotion, we would like to highlight the behavioural consequences of specific negative emotions that often appear in response to threat. In particular experiencing Anger often translates behaviourally to action, diminished risk perception and less careful processing of information. As such, the rapid spread of Anger could have a negative impact on crisis management, even if it could also provide some momentary stress relief at the level of the individual. On the contrary, Fear is linked to avoidance, risk overestimation and increased attention to threat. In theory, collective action can be facilitated by high levels of Anger [65] and low levels of Fear and Sadness [66]. As such, given our results of Anger ‘dominated’ tweets in the #misinformation and a stronger combination of Fear and Sadness in the #Covid-19, we would expect a stronger tendency towards action from the community in the #misinformation subsample. By investigating collective negative emotions, we can gather information on how these emotions may translate into specific actions and the elements that play a key role in the process.

These implications have to be interpreted with some limitations in mind that need to be addressed in future studies. In particular, the availability of adequate training data in the Nordic languages is very scarce. While we could have translated our tweets into English in order to use models that have been trained in English, this would have led to significant loss of information in our data. Instead, we chose to automatically translate the English dataset that we used for the final fine-tuning of a multilingual model into the languages of the study, and then assess the accuracy of such translation with the help of native speakers in each of the Nordic languages. While this approach still carries some loss of information due to inaccuracies in the translation, it is less detrimental and time costly than the alternative solution of translating the entire Twitter dataset into English. Furthermore, our training dataset was unbalanced, containing different amounts of tweets expressing each of the emotions. As such, the model was potentially not able to detect emotions such as Anticipation and Trust and less able to detect e.g., Fear than Anger. Other limitations concern the additional challenges related to Twitter data, such as the characteristically short texts and the use of irony and sarcasm, very present on Twitter during earlier stages of the pandemic at least in other countries [67]. This use of irony and sarcasm is likely to have gone unnoticed and might have resulted in some tweets being erroneously labelled as Joy, when they should have been labelled as Anger. This might also be related to the marked differences in the Finnish data in contrast to the data of the other countries; Finland is the country that differs the most among the Nordic countries present in this study, which makes sense from a geographical and historical perspective and. The same applies to dynamics created by bots; while we consider that tweets created by bots that might have inspired discussion within certain communities are relevant to our study, the bot communities might differ across the Nordic countries. In fact, the Finnish Twittersphere is known to have a specific community of bots [68], which might be related to the differences between the Finnish and the other samples present in our results. Regarding our main findings that hashtagged content carries more negative emotions, we should also keep in mind that the Covid-19 content might be more diluted in non-hashtagged tweets, even if the conditions are still related to Covid-19 due to the high permeability of this crisis in all areas of life.

While our findings are rather intuitive, to our knowledge there is no previous research suggesting that crisis and threat hashtags tend to come accompanied by more negative emotional expression and less positive emotional expression than non-hashtagged content. Given that most previous research on crisis monitoring through Twitter has been done using hashtag-extracted data, highlighting this difference in emotional expression deepens our understanding of how crisis are reflected in social media and where to place the focus during potential panic mitigation strategies. The study leverages on the cross-national aspect of the Covid-19 crisis and the misinformation threat to test the effect of hashtag use on emotional expression based on predictions inferred from the theory of ambient affiliation across countries. This is a unique scenario since other crises tend to be country-specific (e.g., natural disasters, terror attacks, earthquakes and elections) or very extended across time (climate crisis). Our findings are important for the wider research community as they shed light on the interactions between hashtag use and emotional expression. Future studies could further investigate the source of positive emotions during crisis with the aim of amplifying such positive event during crisis management.

## Supporting information

S1 Table Definitions for key concepts framing the research questions. (JPG)

S2 Table Hashtags related to Covid-19 used for filtering tweets into the #Covid-19 subsample. (JPG)

S3 Table Hashtags related to misinformation used for filtering tweets into the #misinformation subsample. (JPG)

S4 Table Translation assessment of training data used in the fine-tuning of the model. (JPG)

S5 Table Performance scores from the XLM-RoBERTa model for emotion detection across the Nordic languages. We adopted a threshold at f1>0.60 indicated by the middle line.
(JPG)

S6 Table Anonymised tweets with high emotionality for the General without hashtag and the #Covid-19. (DOCX)

S7 Table Anonymised tweets scoring high on Anger for the #Covid-19 and the #Disinformation. (DOCX)

S1 Appendix Additional details regarding data collection, data training and data filtering. (DOCX)

S1 Fig Correlations between expression of emotions and number of daily deaths due to Covid-19. The expression of emotions on Twitter are not correlated with fluctuations in the number of daily deaths due to Covid-19 in any of the Nordic countries.
(DOCX)

S2 Fig Distributions of emotional expression across the three different subsamples (Non-hashtagged, #Covid-19 and #misinformation). The distributions of negative emotions saturate on the lower end in the non-hashtagged and #Covid-19 tweets, and the moderate to high ends in the #misinformation tweets. Positive emotions are more equally distributed in the non-hashtagged and #Covid-19 subsamples, but have a distribution clearly pronounced to the lower end in the #misinformation tweets. Fear does not appear to be a driving emotion in any of the conditions, but is concentrated especially in the lower end in the #Covid-19 condition.
(JPEG)
