# Supplementary figures and images for: Emotions on Twitter as crisis imprint in high-trust societies: Do ambient affiliations affect emotional expression during the pandemic?
Source: PLoS One. 2024 Mar 5;19(3):e0296801. doi: 10.1371/journal.pone.0296801 (PMC10914277; doi:10.1371/journal.pone.0296801)

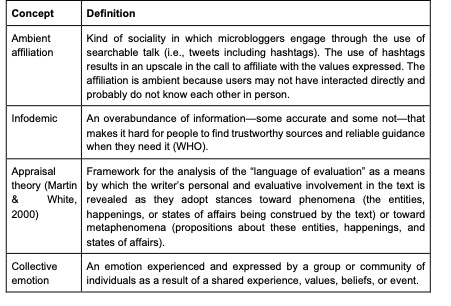

Supplement: S1 Table — (JPG) [file pone.0296801.s001.jpg]

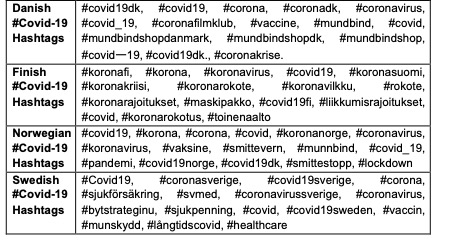

Supplement: S2 Table — (JPG) [file pone.0296801.s002.jpg]

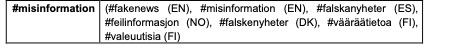

Supplement: S3 Table — (JPG) [file pone.0296801.s003.jpg]

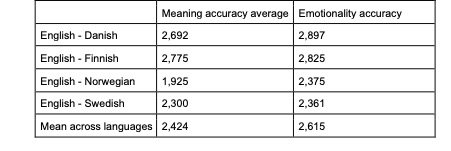

Supplement: S4 Table — (JPG) [file pone.0296801.s004.jpg]

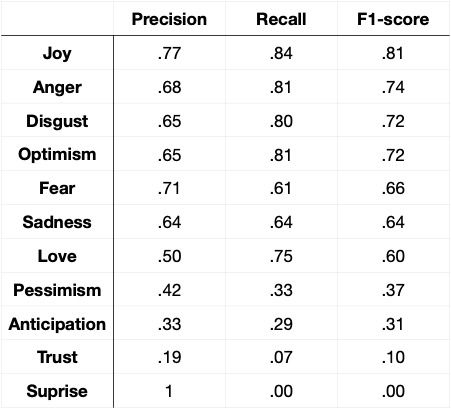

Supplement: S5 Table — We adopted a threshold at f1>0.60 indicated by the middle line. (JPG) [file pone.0296801.s005.jpg]

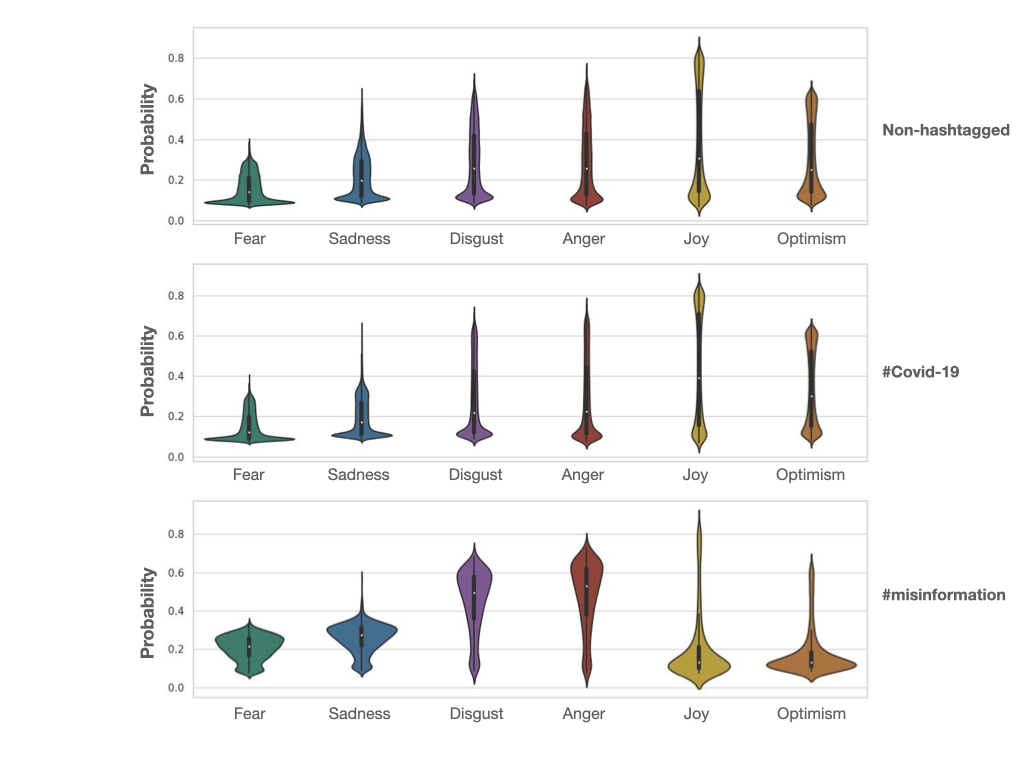

Supplement: S2 Fig — The distributions of negative emotions saturate on the lower end in the non-hashtagged and #Covid-19 tweets, and the moderate to high ends in the #misinformation tweets. Positive emotions are more equally distributed in the non-hashtagged and #Covid-19 subsamples, but have a distribution clearly pronounced to the lower end in the #misinformation tweets. Fear does not appear to be a driving emotion in any of the conditions, but is concentrated especially in the lower end in the #Covid-19 condition. (JPEG) [file pone.0296801.s010.jpeg]
