# Supplementary material for: Emotions on Twitter as crisis imprint in high-trust societies: Do ambient affiliations affect emotional expression during the pandemic?
Source: PLoS One. 2024 Mar 5;19(3):e0296801. doi: 10.1371/journal.pone.0296801 (PMC10914277; doi:10.1371/journal.pone.0296801)
Supplement: S6 Table — (DOCX) [file pone.0296801.s006.docx]

| **General without hashtags** | **Emotionality score** | **#Covid-19** | **Emotionality score** |
| --- | --- | --- | --- |
| '@user: 321 absent?? The vote was for 145 billion. What the f— is this? Am I missing something or is it that we have a bunch of incompetent and lazy citizens in the parliament? https://link_to_video' | 1.9586 | 'Hey @user This is absurd! anger at the vaccine requirement: Very annoying #vaccination https://link_to_video' | 2.2760 |
| The material condition forces nationalism, and we will not be able to escape it. Remember that! The question is therefore about which forms of nationalism our country chooses. Welfare nationalism?  Cultural nationalism?  Chauvinist nationalism? Republican nationalism? ' | 1.8427 | 'So damn tired of this! #professional ban #artist ban #covid #[country] #the government #covid19[country] https://link_to_video' | 2.2726 |
| @user Due to a negative report, IDX fell to about DKK 30. FUV 42. For my part, I consider everything under 42 as cheap now. I own shares so I'm biased!' | 1.9171 | 'Is this really true? If so, it's terrible. Does anyone know? # covid19[country]# [country]pol https://link_to_video' | 2.2722 |
| noted! “screw it and you'll live longer"' | 1.7713 | [Country] has a completely irresponsible attitude to s health checks and ampling - devastating in pandemics and mass migration. I wrote about these shortcomings in January 2018. Why is [country] ruled by useless idiots?? #corona #breakdown https://link_to_video https://link_to_video' | 2.2646 |
| @user: completely out of question to stop the benefit cars. 2% of subsidized cars are in rural areas, 8 out of 10 subsidized drivers are men, high-income earners are subsidized more than low-income earners, free workplace parking is included, which makes subsidized cars extra affordable in metropolitan areas.' | 1.4560 | 'I feel such a deep contempt for our politicians. I am completely amazed how such terribly low-talented people are set to decide over my life and the lives of others. I feel offended and completely trampled on who has to endure this humiliation. # [country]pol # covid' | 2.2676 |

**S6 Table.** **Anonymised tweets with high emotionality for the General without hashtag and the #Covid-19.**
