# Supplementary material for: Emotions on Twitter as crisis imprint in high-trust societies: Do ambient affiliations affect emotional expression during the pandemic?
Source: PLoS One. 2024 Mar 5;19(3):e0296801. doi: 10.1371/journal.pone.0296801 (PMC10914277; doi:10.1371/journal.pone.0296801)
Supplement: S7 Table — (DOCX) [file pone.0296801.s007.docx]

| **#Covid-19** | **Anger score** | **#Misinformation** | **Anger score** |
| --- | --- | --- | --- |
| @user Why not just cancel all departures to these departure points? Or cancel everything? Then you avoid spreading #covid19? #manslaughter #irresponsible | 0.727069 | Again […] #fakenews couldn't be clearer. Their agenda blinds them, forcing them to lie and distort the truth. Shamefully lousy. | 0.727171 |
| Hey @user This is absurd! #vaccination Anger at the vaccine requirement: Exasperating https://link_to_video | 0.7289152741 | #fakenews from @[user] that blue light sabotage would lead to harsh punishments. #[country]pol #löfven #unacceptable | 0.724307 |
| [Country] has a completely irresponsible attitude to health checks and sampling - devastating in pandemics and mass migration. I wrote about these shortcomings in January 2018. Why is [country] ruled by useless idiots?? #[country]pol #corona #breakdown https://link_to_video https://link_to_video | 0.728233 | https://link_to_video @svtnyheter can't present the facts properly when "a black man who is shot by the police in the USA" Then the racism is total in the #redaktionen There will be no prosecution as the police did not commit a crime. #svt #impartiality #fakenews | 0.723803 |
| From 15/3/2020 [country] @user #coronavirus #Covidioten It's so terrible. […] should be in solitary confinement in a secure prison NOW. It's called Genocide https://link_to_video | 0.730306 | If you don't understand this, it's because you only watch […] #fakenews corrupt # lyingmedia #[country)media | 0.719814 |
| Scary lax relationship @user has with lies and concealments! It should be punishable to lie so violently to the Folketing and the population!! #corona[country] #covid19[country]  #[country]pol | 0.728296 | I can’t be bothered to read about #immigration # integrationalleged #racism #corkagegovernment  #fakenews anymore | 0.716662 |

**S7 Table. Anonymised tweets scoring high on Anger for the #Covid-19 and the #Disinformation**
