# Supplementary material for: Emotions on Twitter as crisis imprint in high-trust societies: Do ambient affiliations affect emotional expression during the pandemic?
Source: PLoS One. 2024 Mar 5;19(3):e0296801. doi: 10.1371/journal.pone.0296801 (PMC10914277; doi:10.1371/journal.pone.0296801)
Supplement: S1 Appendix — (DOCX) [file pone.0296801.s008.docx]

| **Stopwords and data collection** |
| --- |
| The tweets in Danish, Norwegian, and Swedish were collected through the HOPE project (<https://hope-project.dk/#/>) before the language tag was available. The stop words were sourced from the Open Subtitles website ([http://www.opensubtitles.org](http://www.opensubtitles.org/)), and generated by selecting the top 100 most frequent words in the four Nordic languages of this study, full lists available at the following [github repository](https://github.com/centre-for-humanities-computing/stopwords-danish-distinct/tree/master/wordlists). For each language, words were cross-checked with the lists from any of the other Nordic languages lists relevant for this study and removed if the word was present across languages. The reason for doing so was to create a list of frequently used words in each of the languages that was still as differentiated as possible from the other Nordic languages partaking in this study. In the case of Norwegian, additional Nynorsk words were added to the Bokmal stop word list after checking for their absence in the other languages. The tweets in Finnish were collected using the equivalent set of stop words in that language. |
| **Training dataset** |
| We used the SemEval 2018, task 1 (1) as the training dataset to finetune the model for emotion detection. This dataset was manually annotated through a crowdsourcing project, with each tweet being annotated by, on average, seven annotators (for further details, see (1)). The labelled emotions were the following: 1) Anger (also includes Annoyance, Rage) 2) Anticipation (also includes Interest, Vigilance) 3) Disgust (also includes Disinterest, Dislike, Loathing) 4) Fear (also includes Apprehension, Anxiety, Terror) 5) Joy (also includes Serenity, Ecstasy) 6) Love (also includes Affection) 7) Optimism (also includes Hopefulness, Confidence) 8) Pessimism (also includes Cynicism, No confidence) 9) Sadness (also includes Pensiveness, Grief) 10) Surprise (also includes Distraction, Amazement) 11) Trust (also includes Acceptance, Liking, Admiration) and, 12) neutral or no emotion. The presence of each of the eleven emotions was analysed for each of the tweets, resulting in a multilabel training dataset in which each tweet can contribute to multiple emotions. The English version of the training dataset consists of 10,983 tweets in total, 6,838 for the training, 886 for the validation and 3,259 for the test set. |
| **Hashtag extraction** |
| In order to create a non-hashtagged, a #Covid-19 and a #misinformation subsample of tweets, we computed hashtag counts to determine which were the most used hashtags in our dataset. For the #Covid-19 subsample, the 16 most often used Covid-19 hashtags were used (**S2 Table**). The number of hashtags was set according to the maximum number of Covid-19 hashtags in the smallest dataset, which was the Norwegian dataset with 16 Covid-19 hashtags. By selecting tweets containing any of these 16 hashtags, we expanded the hashtag sample due to the appearance of co-hashtags. As such the final #Covid-19 subsample contained 8,262 (Danish), 39,045 (Finnish), 5,943 (Norwegian) and 1,062 (Swedish) hashtags. The number of tweets in each language for the #Covid-19 subsample was 21,869 in Danish, 97,277 in Finnish, 7,870 in Norwegian and 21,869 in Swedish. The non-hashtagged subsample was obtained as a random selection of tweets not containing any hashtags, equal in size to the #Covid-19 subsamples for each language (i.e., 21,869 in Danish, 97,277 in Finnish, 7,870 in Norwegian and 21,869 in Swedish). For the #Misinformation subsample, we used misinformation-related hashtags (**S3 Table**). We used both the terms fake news and misinformation translated to the Nordic languages in addition to the English terms, amounting to 354 tweets In Danish, 694 tweets in Finnish, 190 tweets in Norwegian and 579 tweets in Danish. Of note, the definition for disinformation by Buning and colleagues (2) refers to “false, inaccurate or misleading information designed, presented and promoted to intentionally cause public harm or for profit”. We use this term interchangeably with the term “fake news” or “rumour” that has been carefully conceptualised in different studies (3–5). Additionally, we use the term misinformation, in contrast to the term disinformation defined by Buning and colleagues, which makes no assumptions about the intention. As such we aim for a more comprehensive subsample of tweets, possibly containing a wider range of expressed emotion, while still focused on information disorders. The tweet extraction approach in this case did not include the selection through most used hashtags because the prevalence of misinformation related hashtags in Nordic Twitter is very low. Consequently, the number of #misinformation tweets were lower than the number of #Covid-19 tweets. The inclusion of co-occurring hashtags resulted in 305 (Danish), 702 (Finnish), 208 (Norwegian) and 370 (Swedish) hashtags. |

**S1 Appendix. Additional details regarding data collection, data training and data filtering.**

**References**

1. S. M. Mohammad, F. Bravo-Marquez, Emotion Intensities in Tweets. *arXiv:1708.03696 [cs]* (2017) (August 11, 2020).

2. M. de Cock Buning, *A multi-dimensional approach to disinformation: report of the independent High level Group on fake news and online disinformation* (Publications Office of the European Union, 2018).

3. A. Bechmann, B. O’Loughlin, “Democracy and Disinformation: A turn in the debate.”

4. B. Kalsnes, Deciding what’s true: The rise of political fact-checking in American journalism. *Digital Journalism* **6**, 670–672 (2018).

5. E. C. Tandoc, Z. W. Lim, R. Ling, Defining “Fake News”: A typology of scholarly definitions. *Digital Journalism* **6**, 137–153 (2018).
