# Supplementary material for: Emotions on Twitter as crisis imprint in high-trust societies: Do ambient affiliations affect emotional expression during the pandemic?
Source: PLoS One. 2024 Mar 5;19(3):e0296801. doi: 10.1371/journal.pone.0296801 (PMC10914277; doi:10.1371/journal.pone.0296801)
Supplement: S1 Fig — The expression of emotions on Twitter are not correlated with fluctuations in the number of daily deaths due to Covid-19 in any of the Nordic countries. (DOCX) [file pone.0296801.s009.docx]

*
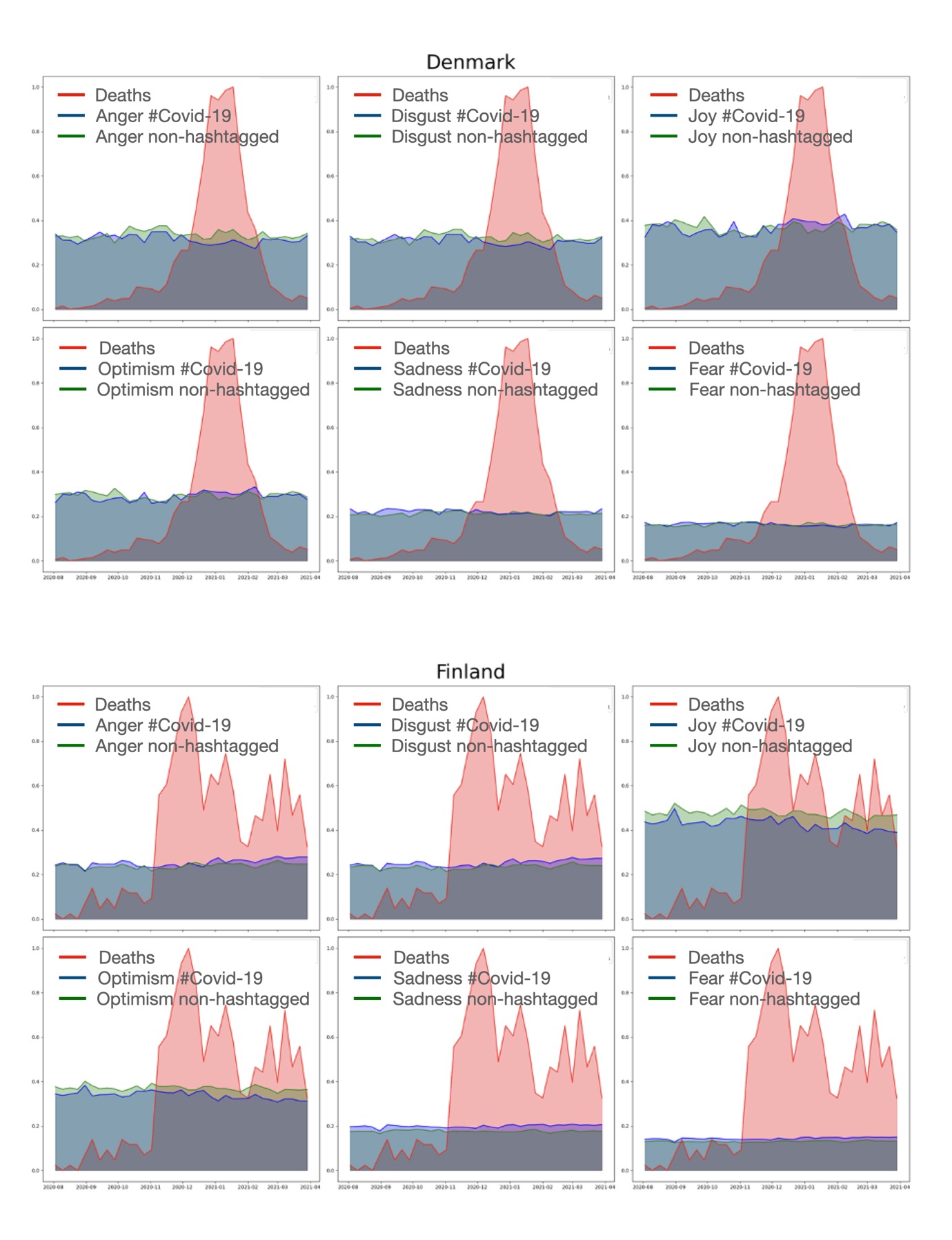
*

**S2 Fig (part 1).** **Correlations between expression of emotions and number of daily deaths due to Covid-19.** The expression of emotions on Twitter are not correlated with fluctuations in the number of daily deaths due to Covid-19 in any of the Nordic countries.

*
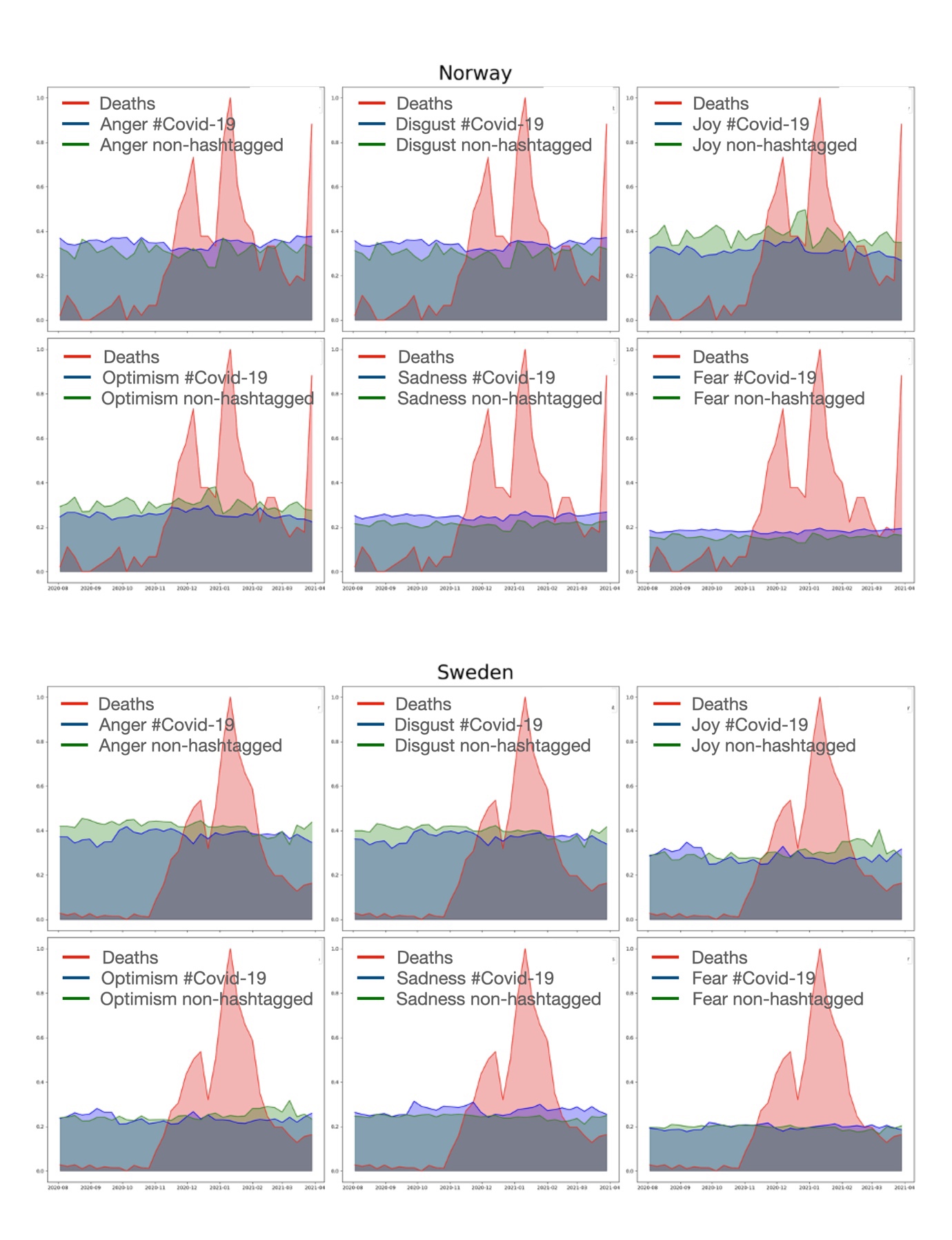
* **S2 Fig (part 2).** **Correlations between expression of emotions and number of daily deaths due to Covid-19.** The expression of emotions on Twitter are not correlated with fluctuations in the number of deaths due to Covid-19 in any of the Nordic countries.
